# Aminolevulinic Acid-Mediated Photodynamic Therapy of Human Meningioma: An *in Vitro* Study on Primary Cell Lines

**DOI:** 10.3390/ijms16059936

**Published:** 2015-04-30

**Authors:** Mustafa El-Khatib, Carolin Tepe, Brigitte Senger, Maxine Dibué-Adjei, Markus Johannes Riemenschneider, Walter Stummer, Hans Jakob Steiger, Jan Frédérick Cornelius

**Affiliations:** 1 Department of Neurosurgery, Universitätsklinikum Düsseldorf, Heinrich Heine Universität, 40225 Düsseldorf, Germany; E-Mails: mustafa.el-khatib@ukb.uni-bonn.de (M.E.-K.); carolin.tepe@uni-duesseldorf.de (C.T.); Brigitte.Senger@med.uni-duesseldorf.de (B.S.); Maxine.Dibue@med.uni-duesseldorf.de (M.D.-A.); Hans-Jakob.Steiger@med.uni-duesseldorf.de (H.J.S.); 2 Center for Molecular Medicine, Universität zu Köln, 50931 Cologne, Germany; 3 Institute for Neurophysiology, Universität zu Köln, 50931 Cologne, Germany; 4 Department of Neuropathology, Regensburg University Hospital, 93042 Regensburg, Germany; E-Mail: markus.riemenschneider@ukr.de; 5 Wilhelm Sander-NeuroOncology Unit, Regensburg University Hospital, 93042 Regensburg, Germany; 6 Department of Neurosurgery, Universitätsklinikum Münster, Westfälische Wilhelms-Universität, 48149 Münster, Germany; E-Mail: walter.stummer@ukmuenster.de

**Keywords:** 5-ALA, aminolevulinic acid, PDT, photodynamic therapy, meningioma, *in vitro*, primary cell culture

## Abstract

Objective: Five-aminolevulinic acid (5-ALA)-induced porphyrins in malignant gliomas are potent photosensitizers. Promising results of ALA-PDT (photodynamic therapy) in recurrent glioblastomas have been published. Recently, 5-ALA-induced fluorescence was studied in meningioma surgery. Here, we present an experimental study of ALA-PDT in an *in vitro* model of primary meningioma cell lines. Methods: We processed native tumor material obtained intra-operatively within 24 h for cell culture. Epithelial membrane antigen (EMA) immunohistochemistry was performed after the first passage to confirm that cells were meningioma cells. For 5-ALA-PDT treatment, about 5000 cells per well were seeded in 20 wells of a blank 96-well plate. Each block of 4 wells was inoculated with 150 µL of 0, 25, 50 and 100 µg/mL 5-ALA solutions; one block was used as negative control without 5-ALA and without PDT. Following incubation for 3 h PDT was performed using a laser (635 nm, 18.75 J/cm^2^). The therapeutic response was analyzed by the water soluble tetrazolium salt (WST-1) cell viability assay 90 min after PDT. Results: 5-ALA-PDT was performed in 14 primary meningioma cell lines. EMA expression was verified in 10 primary cell cultures. The remaining 4 were EMA negative and PDT was without any effect in these cultures. All 10 EMA-positive cell lines showed a significant and dose-dependent decrease in viability rate (*p* < 0.001). Cell survival at 5-ALA concentrations of 12.5, 25, 50 and 100 μg/mL was 96.5% ± 7.6%, 67.9% ± 29.9%, 24.0% ± 16.7% and 13.8% ± 7.5%, respectively. For the negative controls (no 5-ALA/PDT and ALA/no PDT), the viability rates were 101.72% ± 3.5% and 100.17% ± 3.6%, respectively. The LD_50_ for 5-ALA was estimated between 25 and 50 µg/mL. Conclusion: This study reveals dose-dependent cytotoxic effects of 5-ALA-PDT on primary cell lines of meningiomas. Either 5-ALA or PDT alone did not affect cell survival. Further efforts are necessary to study the potential therapeutic effects of 5-ALA-PDT *in vivo*.

## 1. Introduction

Meningiomas account for 20%–34% of intracranial tumors. With gliomas, they are the most common primary intracranial tumors with an annual incidence ranging from two to seven per 100,000 for women, and from one to five for men [1,2,3]. Today, computed tomography (CT) and magnetic resonance imaging (MRI) diagnosis of meningiomas is often incidental, as patients remain asymptomatic for long periods of time due to slow tumor growth [4]. Current standard of care for symptomatic tumors is surgical excision of the tumor—as an alternative for smaller and poorly accessible lesions—radiosurgery. Despite complete resection, recurrence rates of 10%–20% are reported [5,6,7]. Recurrence rates depend on completeness of resection and the World Health Organization (WHO) grade of the meningioma. The recurrence rates of WHO grade III meningiomas are reported to be as high as 80% [8,9]. Conventional radiotherapy and radiosurgery were recommended as adjuvant therapy for incompletely resected, recurrent, atypical or anaplastic meningiomas [10], despite potential serious side effects [3,11,12,13]. Although 80% of all meningiomas may be successfully treated with surgery alone or radiosurgery, five-year control rates for incompletely resected and higher grade meningiomas remain poor after either surgery or radiosurgery [8,11,14]. Therefore, more effective adjuvant therapies without major side effects are required.

Five-aminolevulinic acid (5-ALA) has been under close scrutiny for fluorescence-guided resections because it elicits the accumulation of fluorescing porphyrins in malignant gliomas [15,16,17,18,19,20]. These porphyrins are also phototoxic and have been explored for photodynamic therapy (PDT) of these tumors [18,21,22,23,24,25,26,27,28]. PDT involves the excitation of an administered photosensitizer by light of a specific wavelength to induce energy transfer-producing reactive oxygen species, which then damage the DNA, thereby inducing apoptosis or, at higher doses, necrosis [29].

An endogenous precursor molecule in the heme synthesis, 5-ALA is metabolized to protoporphyrin IX (PPIX). PPIX fluorescence under blue light has been used for intraoperative visualization (photodiagnosis) of malignant gliomas [19]. In addition, PPIX is also a potent photosensitizer, making it useful for PDT [30]. Ferrochelatase, the terminal enzyme of the heme biosynthetic pathway, catalyzes the insertion of iron into PPIX to form heme [31]. Under physiologic conditions, heme regulates its own synthesis via a negative feedback loop inhibiting 5-ALA synthase, the first enzyme in the heme biosynthetic pathway. Administration of excessive amounts of exogenous 5-ALA causes temporary accumulation of PPIX, as ferrochelatase is not immediately capable of converting all the PPIX into heme, thus rendering the cells fluorescent and photosensitive.

Systemic 5-ALA application causes fluorescence of gliomas and also of meningiomas, but not of adjacent brain tissue enhancing visual distinction of tumor tissue from brain tissue. This may help to improve the extent of resection (photodiagnosis) but may also be used for PDT [19,32]. Experimental studies of PDT with 5-ALA confirmed a selective phototoxic effect on glioma cells [21,22] and initial effects on inoperable tumors in patients were reported [23]. While PPIX-PDT appears to be effective in gliomas, the effectivity of 5-ALA-PDT for meningiomas has yet to be established. A previous study comparing ALA-PDT effects between one glioma and one meningioma cell line found a much lower effectiveness in the meningioma cell line [24]. In a more recent study of 5-ALA-PDT in two human meningioma cell lines, the authors found different 5-ALA induced PDT susceptibilities of the cell lines, corresponding to different ferrochelatase activities [33].

To assess whether 5-ALA-induced PDT is a useful tool in therapy of malignant and recurrent meningioma, a better knowledge of PDT susceptibility of different meningioma cell lines is necessary. Therefore, in this study, we evaluated the 5-ALA induced PDT susceptibility of 21 primary meningioma cell cultures.

## 2. Results

### 2.1. Immunohistochemistry

**Figure 1:**
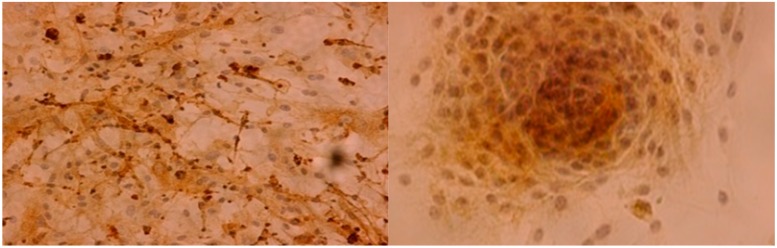
Samples of epithelial membrane antigen (EMA) expression in primary meningioma cell lines; magnification ×200-fold.

Of the 14 cell lines that underwent immunohistochemical detection of epithelial membrane antigen (EMA), ten (71%) were EMA positive and four (29%) were EMA negative (Figure 1). A highly significant (*p* = 0.002) difference of EMA expression depending on the latency of the first passage could be identified, with high EMA expression corresponding to an early passage in average after 7 days (range 5–10 days). Chi^2^-test did not reveal any correlation of EMA expression to WHO grade.

### 2.2. Five-Aminolevulinic Acid (5-ALA) Photodynamic Therapy

**Figure 2:**
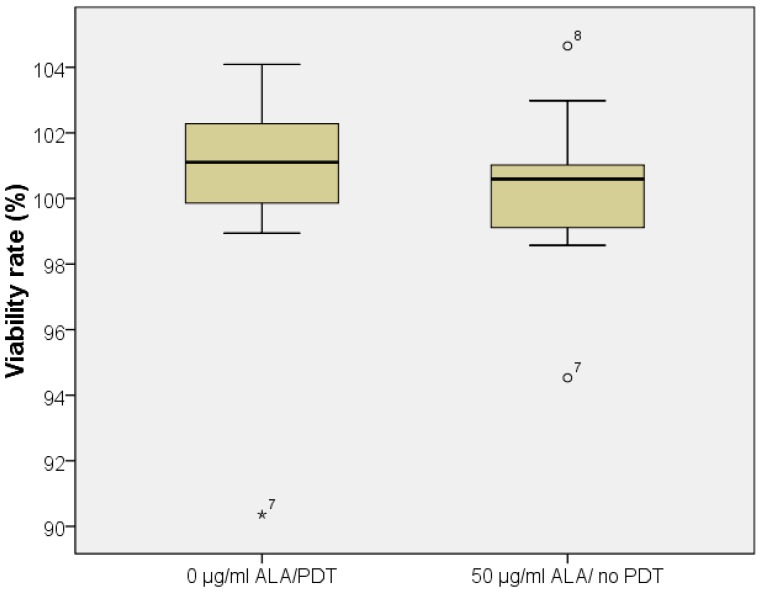
Negative controls; note: outliers (>1 standard deviation) were marked by stars or circles.

Water soluble tetrazolium salt (WST)-1 cell viability assay showed toxicity to the tested cell lines exclusively with the combination of 5-ALA application and laser irradiation. No toxicity (decreased cell viability) was detected in wells containing 50 μg/mL 5-ALA not subjected to irradiation, neither was toxicity detected in wells without 5-ALA subjected to irradiation (Figure 2).

**Figure 3:**
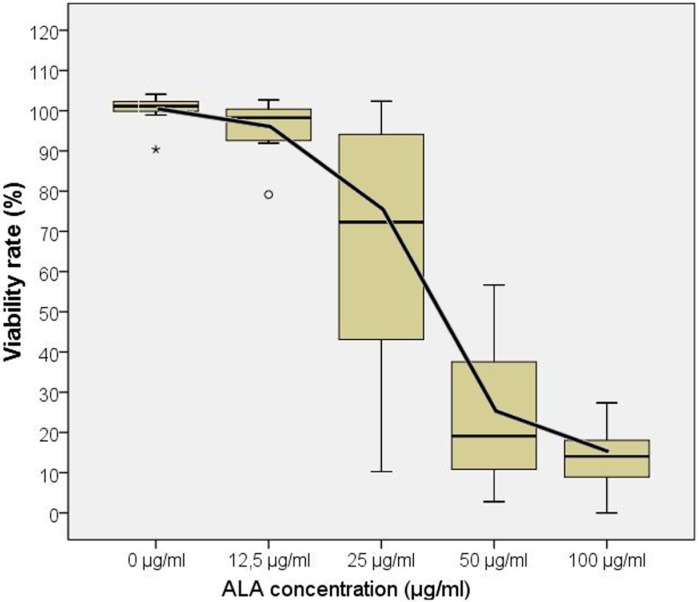
ALA-PDT showed an ALA dose-dependent decrease of cell viability as measured by WST-1 test. Note: the box plots show cell viability rate (%) after 5-ALA-PDT, as measured by WST-1 assay for different 5-ALA concentrations (median ± SD). The graph shows the means of 10 experiments with EMA positive cells. The LD_50_ was found to lie between 25 and 50 µg/mL; note: outliers (>1 standard deviation) were marked by stars or circles.

No toxicity was seen in EMA negative cell lines. Application of 5-ALA and subsequent PDT was found to be toxic in all 10 tested EMA positive cell lines, and toxicity was found to be dose-dependent in all cell lines. Cell survival at 5-ALA concentrations of 12.5, 25, 50 and 100 μg/mL were 96.5% ± 7.6%, 67.9% ± 29.9%, 24.0% ± 16.7% and 13.8% ± 7.5%, respectively. Cell viability decreased significantly (*p* < 0.001) at concentrations 25 μg/mL and above. The viability rates, documented by the WST-1 assay, are demonstrated in Figure 3.

**Figure 4:**
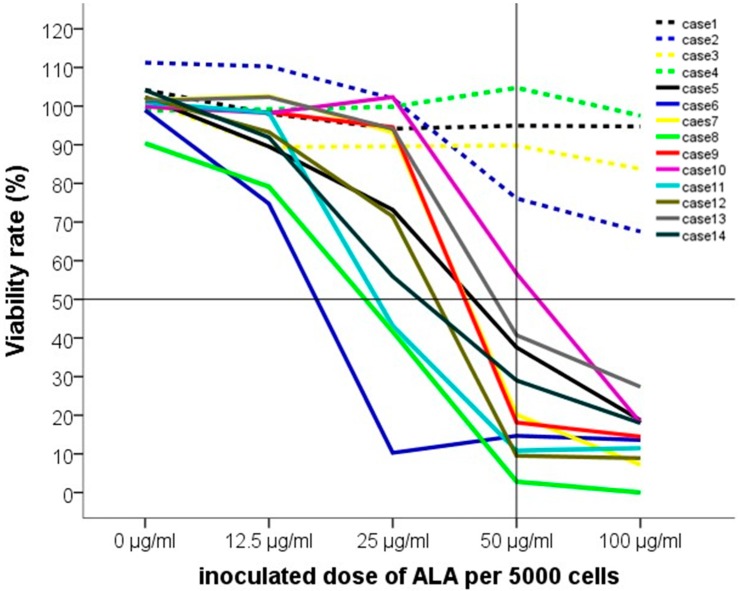
The courses of cell viability in each experiment. Note: the dashed lines show the four EMA negative populations (cases 1–4), the solid lines show the EMA positive cultures (cases 5–14).

Figure 4 demonstrates the course of viability decrease in the ten experiments with EMA-positive cells. There was a significant drop from 25 to 50 µg/mL in all trials (*p* < 0.001). LD_50_ was estimated between 25 and 50 µg/mL. The four EMA negative populations showed no significant effect by 5-ALA-PDT.

## 3. Discussion

In the present study, we demonstrated that 5-ALA-PDT is effective in decreasing viability of primary meningioma cell lines in a dose-dependent manner, with an estimated LD_50_ between 25 and 50 µg/mL. Hefti *et al.* recently reported a very interesting *in vitro* experiment on two immortalized meningioma cell lines (BEN-MEN 1 and HBL-52). Within the same cell lines the results on ALA-PDT were reproducible with a high reliability and validity. However, between the two cell lines, they detected a variable response to the irradiation therapy [33]. We confirmed these results in 10 primary cell lines *in vitro*. All primary cell lines responded positively to ALA-PDT with a significant decrease in viability with increasing concentrations of 5-ALA. The cell lines showed different sensitivities, reflected by the high variance and standard deviation at 25 µg/mL. The significance of this high variance compared to the other used concentrations remains unclear; however, it may reflect the borderline of the LD_50_ between 25 and 50 µg/mL ALA solutions.

Nevertheless, our results confirm Coluccia’s observations *in vivo*. The intraoperative use of 5-ALA for the visualization of meningiomas showed different fluorescent intensity between and within the resected meningiomas [34].

The major determinant of recurrence and prognosis in therapy of meningiomas is the extent of surgical resection of the tumor. Complete resection may be hindered by infiltration or tight contact to dural sinuses, cranial nerves, vessels or brain parenchyma. PDT technically offers a possibility of selective destruction of infiltrating tumor tissue, thereby potentially reducing the rate of recurrence. The first investigations of PDT of meningiomas were carried out in the 1990s [24,35]. Marks *et al.* found dose-dependent toxicity of PDT in five meningioma cell lines and no toxicity in control cell lines. However, the sample size was small and hematoporphyrin derivate (HpD) was used as photosensitizer, which is associated with severe adverse effects and is not tumor specific. Rather, it transgresses the blood-brain barrier along with edema and is thus unspecific for intracerebral tumor tissue [17,18].

A subsequent study of 5-ALA-PDT of immortalized glioma and meningioma cell lines found increased fluorescence and PDT sensitivity of glioma cell lines compared to meningioma cell lines [24]. Although 5-ALA fluorescence guided resection, and PDT is much more established for gliomas than for meningiomas, the potential benefit may be comparable [36,37,38].

Several groups have demonstrated reliable fluorescence of meningiomas after 5-ALA application, which reflects substantial PPIX accumulation [29,32,34,39]. However, Hefti *et al.* revealed inhomogeneity of fluorescence within meningiomas [40], a phenomenon also seen in gliomas [15]. This inhomogeneity may reflect differences in 5-ALA uptake and metabolism. Genetic inhomogeneity, not uncommon for tumors [41,42], may also be a cause of the wide range of 5-ALA-PDT sensitivity found in our experiments. Nevertheless, the mechanisms underlying 5-ALA uptake and regulatory mechanisms of PPIX synthesis have yet to be fully understood. Increased accumulation of PPIX in malignant and pre-malignant tissue compared to healthy tissue may be attributed to either increased 5-ALA uptake, increased rate of conversion of 5-ALA to PPIX or decreased rate of conversion of PPIX to heme [16,43]. Most efforts focused on regulation of ferrochelatase activity and expression, as studies showed an inverse correlation between ferrochelatase expression or activity and PPIX accumulation [9,40,44]. In order to increase photosensitivity of tumors, future efforts should therefore investigate PDT in combination with ferrochelatase inhibitors.

## 4. Experimental Section

### 4.1. Meningiomas

Tumor material used in this study was obtained from 14 patients undergoing surgery for resection of a meningioma. There were five (35.8%) males and nine (64.2%) females. Age at resection ranged from 30 to 82 years with an average of 55.3 years. Twelve were classified as benign (WHO grade I), one as atypical (WHO grade II) and one as anaplastic (WHO grade III) according to the WHO 2007 classification criteria [45]. Informed consent was obtained pre-operatively from each patient.

### 4.2. Cell Culture

Culture of meningioma cells after resection of the tumor was performed by a method adapted from Hardy *et al.* [46]. In preliminary experiments (*n* = 20), we had modified Hardy’s method continuously in order to establish the following procedure in our laboratory: After surgical resection, meningiomas were washed in Hank’s balanced salt solution (HBSS) (GIBCO^®^, Invitrogen, Paisley, UK) and separated from macroscopically visible blood vessels and dura mater, before being minced and centrifuged (500 rpm/400 G). Tissue was then washed again and incubated at 37 °C with 0.05% Trypsin (GIBCO^®^, Invitrogen, Paisley, UK) for 30 min. Medium (DMEM including Glucose and NEAA (GIBCO^®^, Invitrogen, Paisley, UK) with 10% FCS (PAA Laboratories GmbH, Pausching, Austria), 1% glutamax, 1% penicillin, 1% streptomycin and 1% amphotericin B (GIBCO^®^, Invitrogen, Paisley, UK)) were added and the tissue centrifuged again before performing another two washing and centrifugation steps. The obtained cell pellet was re-suspended in medium in a 0.01% collagen type single-coated T25 cell culture flask and cultured as a monolayer. Cells were passaged when a confluence of 70% was reached.

### 4.3. Immunohistochemistry

As culture of primary cells often bears the risk of overgrowth of fibroblasts, we immunohistochemically stained for EMA, which is a specific marker expressed in meningothelial cells. For immunohistochemical detection of EMA, a portion of cells were transferred into a Petri dish containing a cover slip and cultured for several days until the cells had overgrown the cover slip. The cover was then fixed in acetone at −20 °C for 10 min before being left to air drying for 15 min. The dry cover slip was then glued onto a glass slide and was then ready for polymer-conjugated detection of EMA expression by the EnVision™ Detection System (Dako, Glostrup, Denmark). This method involves detection of monoclonal mouse anti-EMA antibodies by binding of peroxidase–polysaccharidpolymer-conjugated anti-mouse secondary antibodies. Firstly, slides were washed in tris buffered saline with tween 20 (TBST, DCS Innovative Diagnostik Systeme, Hamburg, Germany), and then incubated in Dako REAL™ Peroxidase-Blocking Solution (Dako, Glostrup, Denmark) for 10 min. Under subsequent washing, slides were incubated in primary antibody (anti-EMA 1:200 in Dako REAL™ Antibody Diluent (Dako, Glostrup, Denmark)) and then in secondary antibody (EnVision™-Polymer Solution (Dako, Glostrup, Denmark)), each in a humidity chamber for 30 min. The slides were then washed again and incubated in substrate chromogen solution (Dako REAL™ DAB + Chromogen 1:50 in Dako REAL™ Substrate Buffer (Dako, Glostrup, Denmark)) for 4–6 min. After washing in demineralized water, they were contrasted with hemalaun, dehydrated in ethanol and finally mounted with Eukitt^®^ (Sigma-Aldrich, Crailsheim, Germany).

### 4.4. Photodynamic Therapy (PDT)

**Figure 5:**
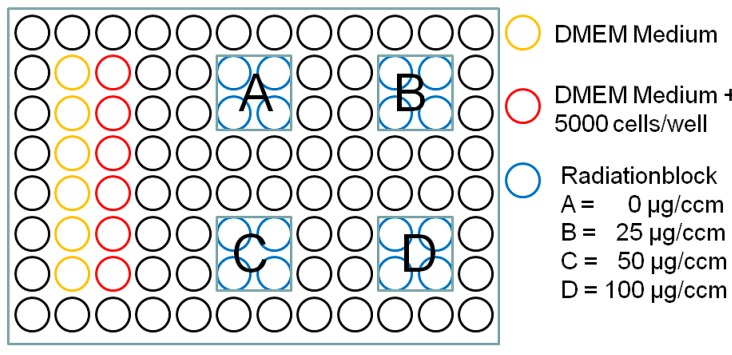
96 well microplate setup.

PDT and control wells were filled with 100 μL of cell solution containing 50 cells per 1 μL medium (counted using trypan blue with PBS 1:2 in a Neubauer counting chamber) corresponding to 5000 cells per well, whereas blank wells were filled with 100 μL of colorless medium. After incubation for 48 h 5-ALA powder (Merck, Darmstadt, Germany) was diluted with aqua dest, in a sterile setup avoiding direct light, to the required concentration, before 50 μL were added to PDT and control wells. Fifty μL of colorless medium were added to blank wells before incubating the microplate in a CO_2_ incubator for 4 h (Figure 5).

**Figure 6:**
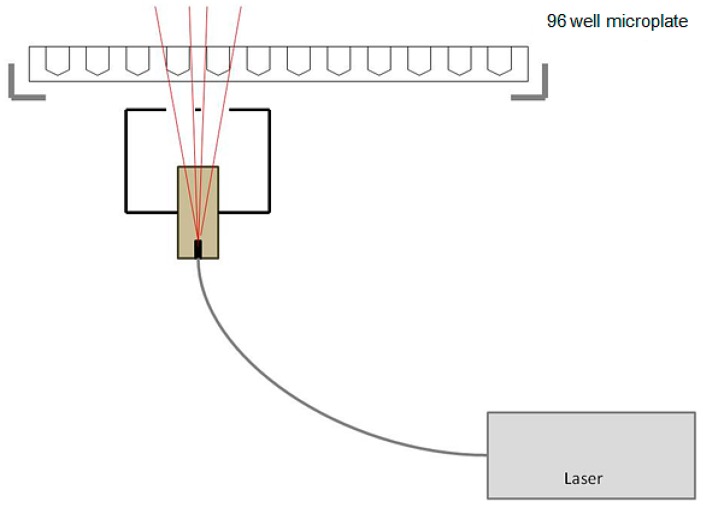
Photodynamic therapy irradiation setup.

The irradiation setup used for PDT has been used in the past for PDT of glioma cell lines in our laboratory and was developed in cooperation with Welabo GmbH (Düsseldorf, Germany). It includes a diode laser (Ceralas 635 Lasersystem, BioLitec GmbH, Jena, Germany), which emits light at a wavelength of 635 nm through a fiberglass probe with a diffusor lens, producing a cone of light. This allows homogeneous irradiation of the wells of the 96-well microplate. Irradiation of the wells by backscatter rays from other wells was avoided by leaving at least two rows of distance between the experimental wells. The light source was arranged 9.3 cm from the microplate in order to achieve an intensity of 30 mW/cm^2^, which was executed for 625 s resulting in a radiation dose of 18.75 J/cm^2^. After irradiation, the microplate was returned to the incubator for 90 min before performing the cell viability assay (Figure 6).

### 4.5. WST-1 (Water Soluble Tetrazolium Salt) Cell Viability Assay

Standard WST-1 assay (protocol and reagents Roche Diagnostics GmbH, Mannheim, Germany), which is based on living cells’ capacity to convert red tetrazolium salt into dark red formazan, which can be photometrically quantified, was used to determine cell viability after PDT.

### 4.6. Data Analysis

SPSS Statistics 21 (IBM Corporation, Somers, CT, USA), was used to perform Chi^2^-test, student’s *t*-test, Kruskal–Wallis-Test and one-way ANOVA, in which *p* <0.05 and *p* < 0.01 were considered significant and highly significant, respectively. Data was expressed as average ± standard deviation. Graphs were created using SPSS.

## 5. Conclusions

Photodynamic therapy using the photosensitizer 5-ALA is effective in a dose-dependent manner in primary human meningioma cell lines. Further investigations are necessary to understand the transduction pathway of 5-ALA in tumor cells and to understand variation between individual meningiomas. This may open the possibility of clinical trials to test the therapeutic effect of ALA-PDT *in vivo* in meningiomas.
